# Towards FUS lung cancer ablation: the lung flooding process from a physiological and physical view point

**DOI:** 10.1186/2050-5736-3-S1-P78

**Published:** 2015-06-30

**Authors:** Frank Wolfram, Thomas G Lesser

**Affiliations:** 1 SRH Waldklinikum Gera, Gera, Germany

## Background/introduction

Unilateral lung flooding replaces air with saline in lung parenchyma. It has been shown, that in flooded condition ultrasound guidance and HIFU ablation of central lung cancer tissue is feasible. The flooding process generates a saline-lung compound which is different than known parenchymal tissue. Complete understanding of the flooding process is essential for its implementation in a HIFU cancer ablation scheme.

Therefore a detailed excurse of the flooding mechanism and its influences in acoustic and physiological conditions will be discussed. However, before initiating the first human pilot, several issues remain. So far the usability of MR guiding and aspects of HIFU effects on flooded lung parenchyma are unknown.

## Methods

Human lung lobes, containing Non Small Cell Lung Cancer (NSCLC), are resected after complete intra-surgical atelectasis. The lobes are flooded *in vitro* with degased saline under static pressure of 30 cm water column. Images based on T1w, T2w sequences were acquired by MRI (Achieva 1.5T, Phillips, The Netherlands). A broadband acoustic immersion technique is used to determine the attenuation properties of flooded lung parenchyma. *In vivo* lung flooding was performed in a porcine large animal model “deutsches Landschwein” ca. 30kg. For ventilation a model specific double lumen catheter was trans-bronchially inserted. After 30min FIO 1.0 oxygen ventilation, the left lung wing was flooded with saline under static pressure of 30cm H_2_O column. Flooding was maintained for 90 min under continuous monitoring of vital parameters (SO2, pCO2, pAp, HR).

## Results and conclusions

Flooding was performed successful and all animals (4/4) survived the procedure. Confirmed by Ultrasound B Mode, Lung parenchyma showed no residual gas content. The flooding procedure was stable over 90min, which is a sufficient treatment window for FUS interventions. Lung cancer tissue (NSCLC) could be well demarked from flooded lung in T1 and T2 weighted images. These preliminary results indicate that MR guidance in flooded lung is feasible. The attenuation of flooded lung parenchyma was estimated to be 0,12 dB/cm/MHz, which serves as a superior acoustic path. A review of published lung flooding procedures (perfluorcarbone, saline) will be discussed regarding its safety and usability for FUS lung cancer treatment. Further capabilities of lung-flooding will be demonstrated based on the animal model.

**Figure 1:**
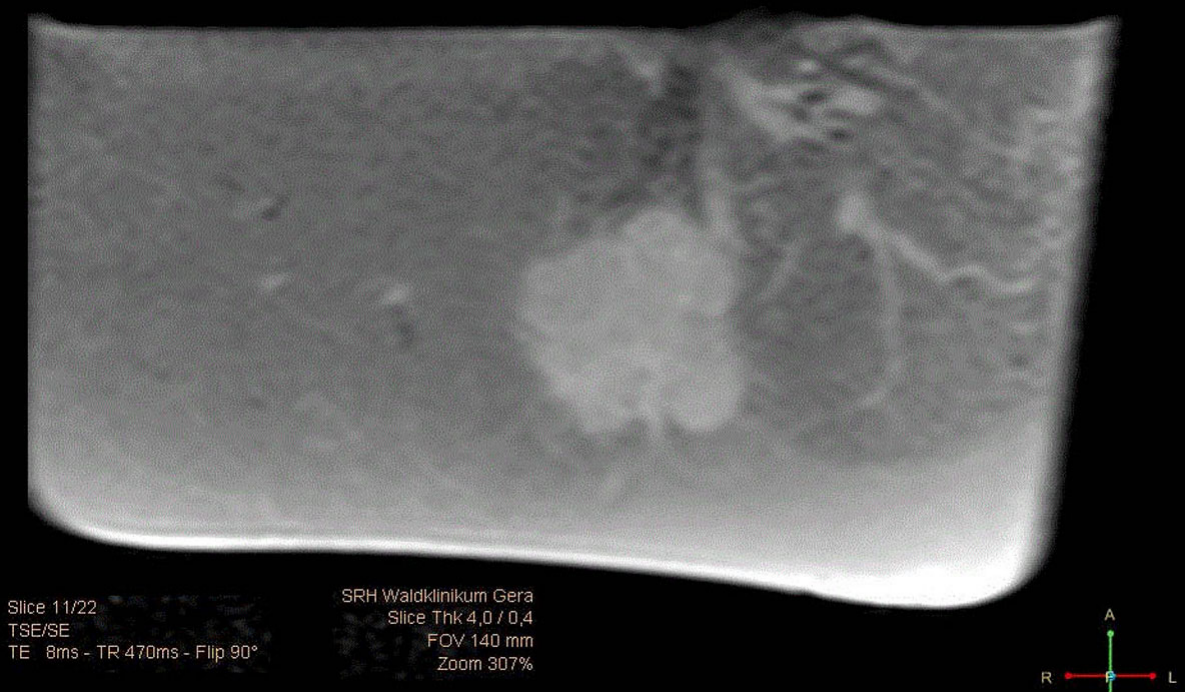
Flooded lung lobe containing NSCLC in T1w MR image

**Figure 2:**
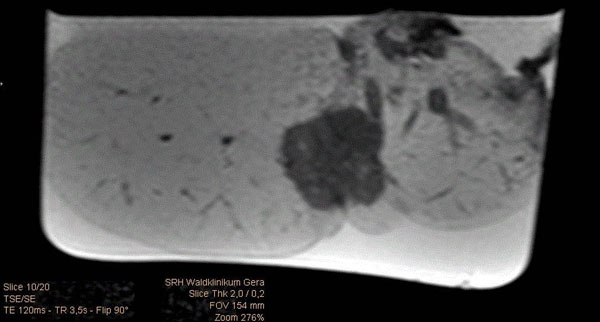
Flooded lung lobe containing NSCLC in T2w MR image

